# **β**-Adrenergic Receptor-Stimulated Cardiac Myocyte Apoptosis: Role of **β**1 Integrins

**DOI:** 10.1155/2011/179057

**Published:** 2011-05-24

**Authors:** Parthiv Amin, Mahipal Singh, Krishna Singh

**Affiliations:** Department of Physiology, James H Quillen Veterans Affairs Medical Center, James H Quillen College of Medicine, East Tennessee State University, P.O. Box 70576, Johnson City, TN 37614, USA

## Abstract

Increased sympathetic nerve activity to the myocardium is a central feature in patients with heart failure. Accumulation of catecholamines plays an important role in the pathogenesis of heart disease. Acting via *β*-adrenergic receptors (*β*-AR), catecholamines (norepinephrine and isoproterenol) increase cardiac myocyte apoptosis *in vitro* and *in vivo*. Specifically, *β*_1_-AR and *β*_2_-AR coupled to G*α*s exert a proapoptotic action, while *β*_2_-AR coupled to Gi exerts an antiapoptotic action. *β*1 integrin signaling protects cardiac myocytes against *β*-AR-stimulated apoptosis *in vitro* and *in vivo*. Interaction of matrix metalloproteinase-2 (MMP-2) with *β*1 integrins interferes with the survival signals initiated by *β*1 integrins. This paper will discuss background information on *β*-AR and integrin signaling and summarize the role of *β*1 integrins in *β*-AR-stimulated cardiac myocyte apoptosis.

## 1. Introduction

Heart failure is a leading cause of morbidity and mortality in the western world. Cardiac myocytes are generally viewed as terminally differentiated and incapable of reentering the cell cycle. Myocyte apoptosis is shown to occur in human myocardium and animal models of cardiovascular disease under a variety of pathological states of the heart [1–4]. The apparently limited capacity for regeneration of myocytes in the adult heart suggests that cardiac myocyte loss due to apoptosis may contribute to the progression of heart failure. 

Increased sympathetic nerve activity is a central feature in patients with heart failure [5]. Initial release of catecholamines by the sympathetic nervous system exerts important tonic effects on the biology of cardiac myocytes leading to increased contractility. However, chronic increase in sympathetic activity is known to have adverse effects in the heart. The efficacy of *β*-AR antagonist in improving the clinical outcome as well as benefit in long-term morbidity and mortality of patients with chronic heart failure [6] has confirmed the importance of sympathetic nerve activity in the pathological remodeling, a process that leads to progressive left ventricular (LV) dilation and contractile dysfunction. Norepinephrine (NE), a primary neurotransmitter of sympathetic nervous system, signals via its interaction with *α*- and *β*-adrenergic receptors (ARs), a family of G protein-coupled receptors (GPCRs). Specific stimulation of *β*_1_-AR using NE or isoproterenol (*β*-AR agonist) induces apoptosis in cardiac myocytes *in vitro* and *in vivo* [7–9]. Increased myocyte apoptosis may influence the development of heart failure. 

Integrins are a large family of heterodimeric transmembrane receptors composed of *α* and *β* subunits. Integrins play a significant role in cell-matrix interactions. They are involved in a variety of functions like gene expression and regulation, organogenesis, cell proliferation, differentiation, migration, and death. In the heart, integrins are shown to regulate cellular phenotype in the developing and postnatal myocardium. They also serve as mechanotransmitters during normal development and in response to physiological and pathophysiological signals [10–12]. Cardiac myocytes predominantly express *β*1 integrins. *β*1 integrins play an important role in *β*-AR-stimulated LV remodeling with effect on myocyte apoptosis [9]. 

This paper will discuss (a) the expression of AR subtypes in the heart and their role in cardiac myocyte apoptosis, (b) molecular signals involved in *β*-AR-stimulated myocyte apoptosis, and (c) role of *β*1 integrins in the regulation of *β*-AR stimulated cardiac myocyte apoptosis.

## 2. Adrenergic Receptors (ARs) and Cardiac Myocyte Apoptosis

### 2.1. AR in Cardiac Myocytes

Nine subtypes of AR have been identified [13]. Cardiac myocytes express at least six subtypes of AR which include three subtypes of *β*-AR (*β*_1_, *β*_2_, *β*_3_) and three subtypes of the *α*_1_-AR (*α*_1A_, *α*_1B_, and *α*_1C_) [14, 15]. Cardiac myocytes do not appear to express *α*_2_-AR in most species [16]. Based on receptor number, *β*_1_-AR generally predominates, although the ratio of *β*_1_ to *β*_2_-AR varies among species and under various pathophysiological states [6]. 

Both *β*_1_- and *β*_2_-AR couple to the stimulatory G protein (G*α*s). This coupling normally leads to the activation of adenylyl cyclase, production of cAMP, and activation of protein kinase A (PKA). *β*_2_-AR also couples to Gi, a different heterodimeric G-protein whose G*α* subunit can inhibit adenylyl cyclase, thereby inhibiting synthesis of cAMP and activation of PKA. This coupling may require phosphorylation of *β*_2_-AR by cAMP-dependent protein kinase A [17]. The dual coupling of *β*2-AR may induce compartmentalization of G*α*s-stimulated cAMP signal affecting only plasma membrane effectors such as L-type calcium channel, without affecting cytoplasmic target proteins such as phospholamban and myofilament contractile proteins [18]. In normal myocytes, *β*_2_-ARs are localized exclusively to the deep transverse tubules, whereas *β*_1_-ARs are distributed across the whole cell surface. Redistribution of *β*_2_-AR from transverse tubules to the cell crest occurred in myocytes isolated from a rat model of heart failure. This redistribution led to cAMP signals in both cell crest and T-tubule, a pattern similar to that observed for *β*_1_-AR stimulation [19]. Thus, the selective activation of *β*_2_-AR-Gi pathway, redistribution of *β*_2_-AR during heart failure, and changes in the compartmentalization of cAMP may have implications during the development of heart failure. 

Pharmacological approaches have suggested that *β*_3_-AR and a putative *β*_4_-AR are present in rodent heart [20, 21]. Their role in cardiac myocyte apoptosis remains to be investigated. Of note, activation of the *β*_3_-AR pathway by catecholamines may contribute to the myocardial dysfunction during sepsis [22], and putative *β*_4_-AR may represent propranolol-insensitive state of *β*_1_-AR [23, 24]. 

### 2.2. *β*-AR in Cardiac Myocyte Apoptosis

Chronic exposure to catecholamines is known to be toxic to cardiac myocytes [25]. Tonic exposure of feline cardiac myocytes to NE caused spontaneous contractions followed by hypercontracture, leading to cell death [26]. This effect was prevented by *β*-AR antagonist propranolol, but not by *α*-AR antagonist, and was mimicked by *β*-AR agonist isoproterenol. Communal et al. provided evidence that stimulation of *β*-AR increases apoptosis in adult rat ventricular myocytes (ARVM) [27]. Similar observations were made in neonatal rat ventricular myocytes and in the myocardium of rats and mice treated with isoproterenol [7, 9, 28, 29]. Interestingly, *β*-AR blockers such as metoprolol and carvedilol reduced myocyte apoptosis and improved cardiac systolic function in animal models of chronic heart failure [30, 31]. These studies suggest that increased sympathetic activity contributes to myocardial failure, at least in part, via *β*-AR-stimulated apoptosis of cardiac myocytes.

Pharmacologic manipulations indicated that specific stimulation of *β*_1_-AR induces proapoptotic signals, while specific linkage of *β*_2_-AR to Gi plays an antiapoptotic role in ARVM [32, 33]. Transgenic mice studies in the heart concur with these observations to some extent. Transgenic mice overexpressing *β*_1_-AR exhibited increase in basal contractile function at young age. This difference in contractility was lost at 16 weeks (age), and contractility continued to decline thereafter, going to less than 50% of wild-type value at 35 weeks [34]. This decline in contractility associated with increased cardiac myocyte apoptosis and expression of pro-apoptotic protein Bax [35]. Transgenic mice overexpressing *β*_2_-AR (60–100-fold over the endogenous level) in the myocardium show enhanced cardiac function without deterioration into heart failure [36–39]. In contrast, higher levels (200–350-fold over the endogenous level) resulted in age-dependent progression to cardiomyopathy which associated with LV dilation, fibrosis and decreased contractility [39]. These deleterious effects of *β*2-AR overexpression could be attributed to the enhanced coupling of *β*2-AR to G*α*s. It is interesting to note that *β*2-AR overexpression (200-fold over the endogenous level) preserved LV contractility in a mouse model of myocardial infarction while exhibiting similar cardiac hypertrophy and chamber size as wild-type mice [40]. Overexpression of G*α*s also had deleterious effects on the heart with chamber dilation, reduced ejection fraction, and increased myocardial fibrosis and myocyte apoptosis [41, 42]. In contrast, targeted inhibition of Gi signaling in the heart worsens the outcome after myocardial ischemia with increased myocyte apoptosis, suggesting a role for Gi in cell survival [43].

### 2.3. Molecular Signals Involved in *β*-AR-Stimulated Apoptosis

*β*-AR-stimulated apoptosis is influenced by the intracellular and extracellular signals. With respect to the intracellular signals, the apoptotic pathway (*β*1-AR-G*α*s) involved activation of PKA [32, 33], while the survival signaling pathway (*β*_2_-AR-Gi) involved activation of phosphatidylinositol 3-kinase (PI3-kinase) and Akt [18]. However, transgenic mice studies question the involvement of PKA in *β*-AR-stimulated apoptosis. This is based on the findings that prolonged *β*-AR stimulation may decrease the levels of *β*_1_-AR and G*α*s may become uncoupled from adenylyl cyclase, leading to activation of calcium/calmodulin kinase II (CaMKII) [44]. Using two genetically defined *β*1-AR systems (adult cardiac myocytes isolated from *β*_2_-AR knockout mice and adenovirus-mediated transfer of the mouse *β*_1_-AR in myocytes isolated from *β*_1_-AR and *β*_2_-AR double knockout mice), it was demonstrated that sustained *β*_1_-AR stimulation delivers a powerful cardiac apoptotic signal via a CaMKII-dependent, rather than a PKA-dependent, mechanism [45]. Recent studies using *β*_1_-AR knockout mice demonstrated that *β*_1_-AR stimulates CaMKII and enhances cardiac dysfunction after myocardial infarction [46]. Cardiac myocytes express delta isoform of CaMKII. Adenoviral-mediated expression of constitutively active CaMKII(deltaC) increased cardiac myocyte apoptosis which associated with increased mitochondrial cytochrome *c* release. The increase in cardiac myocyte apoptosis and cytochrome *c* release was attenuated by coexpression of anti-apoptotic protein Bcl-X(L). Furthermore, expression of a dominant negative mutant of CaMKII(deltaC) not only prevented CaMKII(deltaC)-mediated apoptosis but also protected cells from multiple death-inducing stimuli [47]. 

Mitogen-activated protein kinases (MAPKs), a large family of serine-threonine kinases, have important functions as mediators of intracellular signal transduction. Three subgroups of MAPKs have clearly been identified: c-jun N-terminal kinases (JNKs), p38 kinase, and ERK1/2. *β*-AR stimulation has been shown to activate these three subgroups of MAPKs [48]. Activation of JNKs plays a pro-apoptotic role [49]. Superoxide dismutase/catalase-mimetics or catalase overexpression inhibited JNK activation and *β*-AR-stimulated apoptosis. Inhibition of mitochondrial permeability transition pore opening or caspase activation also decreased *β*-AR-stimulated apoptosis [49]. These studies suggested that *β*-AR-stimulated apoptosis in ARVM involves reactive oxygen species/JNK-dependent activation of the mitochondrial death pathway. In this pathway, small GTPase Rac 1 may act upstream in the activation of JNKs [50]. Studies from our lab have shown that *β*-AR stimulation activates glycogen synthase kinase-3*β* (GSK-3*β*), and activation of GSK-3*β* plays a pro-apoptotic role in *β*-AR-stimulated apoptosis via the involvement of mitochondrial death pathway [51]. Adenoviral-mediated overexpression of constitutively active GSK-3*β* increased JNK phosphorylation, suggesting that GSK-3*β* may also act upstream in the activation of JNKs [52]. Further investigations are needed to clarify the sequence of events involved in the activation of JNK and its linkage with mitochondrial death pathway.

Inhibition of ERK1/2 using PD98059 had no effect on *β*-AR-stimulated apoptosis. On the other hand, SB-202190, an inhibitor of p38 kinase, potentiated *β*-AR-stimulated apoptosis in ARVM, suggesting a protective role for p38 kinase [48]. Pharmacological approach coupled both *β*_1_- and *β*_2_-AR to the activation of p38 kinase, although *β*-AR-stimulated activation of p38 kinase could be inhibited by inactivation of Gi using pertussis toxin. In adult mouse myocytes, *β*_2_-AR activated p38 kinase independent of Gi [53]. Transgenic mice studies uncovered an apoptotic role for p38 kinase, specifically for isoform *α*. Inhibition of p38 kinase(*α*), by mating mice expressing dominant negative p38 kinase(*α*) with mice overexpressing *β*_2_-AR, reversed depressed LV function and reduced apoptosis in mice overexpressing *β*_2_-AR. Inhibition of p38 kinase(*α*) had no effect on *β*1-AR overexpressing mice [54]. These transgenic mice studies suggest that p38 kinase(*α*) plays a pro-apoptotic role during the development of cardiomyopathy following chronic *β*_2_-AR stimulation. These differential effects of p38 kinase may reflect nonspecific effects of pharmacological inhibitor and/or overexpression of *β*_2_-AR. It may also reflect acute versus chronic stimulation of *β*-AR. The transgenic mice studies were carried out in 11–14-months old mice versus acute stimulation of *β*-AR *in vitro*.

Extracellular signals also modulate *β*-AR-stimulated cardiac myocyte apoptosis. Matrix metalloproteinases (MMPs), a large family of endopeptidases, have the ability to degrade extracellular matrix (ECM) proteins, and therefore, play a fundamental role in tissue remodeling, including the heart [55–57]. Tissue inhibitors of MMPs (TIMPs) inhibit MMPs activity by binding to the active site. The involvement of MMP-2 is considered important since MMP-2 is capable of degrading elastin as well as interstitial fibrillar collagen. These effects of MMP-2 can ultimately lead to systolic and diastolic impairment of the heart. Treatment of cardiac rings with active MMP-2 decreased cardiac tissue tensile strength and caused systolic and diastolic dysfunction [58]. Cardiac-specific expression of MMP-2 induced the development of cardiac contractile dysfunction in the absence of superimposed injury [59]. Targeted deletion of MMP-2 attenuated early rupture and improved fractional shortening in mice after myocardial infarction [60]. Our laboratory has provided evidence that *β*-AR stimulation (isoproterenol, 24 h) increases expression of MMP-2 and TIMP-1, and decreases expression of TIMP-2 in ARVM [61]. *β*-AR stimulation had no effect on the expression or activity of MMP-9. Inhibition of MMPs using GM-6001 (a broad-spectrum inhibitor of MMPs), SB3CT (inhibitor of MMP-2), or purified TIMP-2 (tissue inhibitor of MMP-2) inhibited *β*-AR-stimulated apoptosis in ARVM. This decrease in apoptosis associated with inhibition of JNK activity decreased cytosolic cytochrome *c* levels and maintenance of mitochondrial membrane potential. On the other hand, treatment with active MMP-2 alone increased cytosolic cytochrome *c* levels and the number of apoptotic cardiac myocytes [61, 62]. These studies highlight the importance of MMP-2 in *β*-AR-stimulated cardiac myocyte apoptosis and provide evidence that MMP-2 is capable of modulating JNK-dependent mitochondrial death pathway.

Recently, our laboratory has identified ubiquitin in the conditioned media of ARVM. Stimulation of *β*-AR increased levels of extracellular ubiquitin in the media. Treatment of ARVM using purified ubiquitin inhibited *β*-AR-stimulated apoptosis. This inhibition of apoptosis associated with inactivation of GSK-3*β*/JNK and mitochondrial death pathways [52]. Growing evidence suggests that while formation of multiubiquitin chains targets proteins for destruction by the proteasomal complex, monoubiquitination mediates more diverse functions such as protein transport and transcription regulation [63–65]. Using methylated ubiquitin, incapable of forming polyubiquitin chains, it was demonstrated that the anti-apoptotic effects of extracellular ubiquitin are exerted by monoubiquitination of cellular proteins [52]. 

## 3. Integrins and Heart: General Concepts

Integrins link the ECM proteins and the intracellular cytoskeleton. Integrins consist of *α* and *β* subunits, with *α* subunits ranging from 120 to 180 kDa, while *β* subunits range from 90 to 110 kDa [66, 67]. Integrin subunits consist of large extracellular domain (700–1100 amino acids), a single transmembrane segment, and short cytoplasmic tails, ranging from 20 to 60 amino acids [68]. Integrins are bidirectional signaling molecules. The extracellular binding activity of integrins is regulated from intracellular signals (inside-out signaling). Through inside-out signaling, integrins can undergo a switch from a low affinity/avidity state to a high affinity/avidity state. On the other hand, binding of integrins to ECM proteins results in intracellular signaling events. When the extracellular domain of integrin receptor becomes occupied by ligand, the integrins set off a cascade of events termed “outside-in” signaling. This may result in modifications of intracellular pH and cytosolic calcium, and activation of intracellular signaling kinases, leading to alterations in cell morphology, migration, proliferation, differentiation, survival, suppression of tumorigenicity and so forth [69–71]. 

A significant role of integrins in the heart is their ability to serve as mechanotransducers during normal development and in response to physiological and pathophysiological signals [72]. Mechanical stimulation as well as a variety of growth factors like platelet-derived growth factor, insulin-like growth factor, angiotensin II and transforming growth factor-*β* modulate the expression of several integrins as well as specific ECM components such as interstitial collagens, osteopontin, fibronectin and laminin. The myocyte integrin-ECM interactions may play a fundamental role in the pumping function of heart. The ECM surrounding the individual myocytes coordinates the transduction of force to the whole ventricular chamber, so that the heart can function as a single pump [73]. These mechanical linkages (integrin-ECM) also prevent myocyte slippage during contraction. Disruption of the linkages is suggested to occur during the transition from compensated to decompensated heart failure in animal model [74] and in patients with tachycardia-induced heart failure [75]. This disruption may lead to release of cardiac myocytes from their ECM attachment sites, resulting in apoptosis. This process is called anoikis (Greek for homelessness) [76]. It was proposed to be responsible for selective myocyte death due to apoptosis in the heart [74]. The mechanisms involved in the disruption of these linkages are not clearly understood. However, it may involve shedding or cleavage of the extracellular domain (involved in the binding with ECM proteins) of integrins due to a class of enzymes called shedases that include A disintegrin and metalloproteinases (ADAMs) and MMPs [77–79]. The shedding of extracellular domain of *β*1 integrin may subject the cell to altered mechanical force that can be detrimental to the long-term cell survival. Shedding of *β*1 integrins is described in the heart during the transition from cardiac hypertrophy to heart failure [74]. Chronic stimulation of *β*-AR induces *β*1 integrin shedding in the mouse heart [80]. 

Cardiac myocytes predominantly express *β*1 integrins. However they express *β*1D, a differentially spliced variant of *β*1 integrin. *β*1D has a unique cytoplasmic domain of 50 amino acids with the last 24 amino acids encoded by an additional exon. However, both integrin isoforms, *β*1A and *β*1D, were found to be functionally similar with regard to integrin signaling [81, 82]. In the myocytes, *β*1 integrins can heterodimerize with integrin *α* subunits (*α*1, *α*3, *α*5, *α*6, and *α*7b) [83]. Due to the predominant expression of *β*1 integrins in myocytes, the paper summarizes the role of *β*1 integrins in *β*-AR-stimulated apoptosis.

## 4. Cross-Talk between **β**1 Integrins and **β**-AR: Role in Cardiac Myocyte Apoptosis

Integrins can themselves signal through a host of pathways. However, integrins are capable of collaborating with growth factors and their receptors leading to changes in intracellular signals. Many studies now indicate that *β*1 integrins alter AR signaling and influence myocyte phenotype with respect to hypertrophy and apoptosis. *β*1 integrins participate in *α*_1_-AR-induced hypertrophy of neonatal rat cardiac myocytes [84, 85]. A gene expression profile of the myocardial response to clenbuterol, a *β*_2_-AR agonist shown to induce cardiac hypertrophy, demonstrated upregulation of genes associated with integrin-mediated cell adhesion and signaling [86]. Using Cre-Lox technology to inactivate the *β*1 integrin gene exclusively in cardiac myocytes, Shai et al. [87] demonstrated that *β*1 integrins play an important role in myocardial fibrosis and cardiac failure [87]. *In vitro*, stimulation of *β*1 integrin signaling using laminin or adenoviral-mediated overexpression of *β*1A integrin protected ARVM from *β*-AR-stimulated apoptosis [62, 88]. This decrease in apoptosis associated with decreased cytosolic cytochrome *c* levels. On the other hand, expression of a cytoplasmic domain of *β*1 integrin, present as a result of integrin shedding, induced apoptosis in ARVM [80]. This induction of apoptosis associated with activation of caspase-8, Bid cleavage, decreased mitochondrial membrane potential and increased cytosolic cytochrome *c* suggesting involvement of caspase-8 and mitochondrial death pathway. Deficiency of *β*1 integrins as demonstrated using *β*1 integrin heterozygous knockout mice associated with increased cardiac myocyte apoptosis in the heart after myocardial infarction and isoproterenol infusion [9, 89]. *β*1-integrin-deficient mice exhibited enhanced LV dysfunction and dilation after myocardial infarction when compared to the wild-type mice. Myocyte cross-sectional area (a measure of myocyte hypertrophy) and myocardial fibrosis were significantly lower in *β*1-integrin-deficient mice after chronic *β*-AR stimulation [9]. Thus, *β*1 integrins influence *β*-AR responsiveness and play a crucial role in *β*-AR-stimulated myocardial remodeling with effects on cardiac myocyte hypertrophy and apoptosis. Increased cardiac myocyte apoptosis and decreased myocardial hypertrophy and fibrosis during *β*1 integrin deficiency may induce LV dilation due to side-to-side slippage of myocytes during deficiency of *β*1 integrins. It is interesting to note that a combined deficiency of dystrophin and *β*1 integrins in cardiac myocytes decreased ventricular function and blunted adrenergic responsiveness [90]. Thus, *β*1 integrin signaling has the potential to negate the apoptotic effects of *β*-AR stimulation in cardiac myocytes. 

Integrin engagement with ligands initiates autophosphorylation of focal adhesion kinase (FAK) at Tyr397. This autophosphorylation site provides a binding site for Src, which phosphorylates FAK at Tyr576 and Tyr577 to further activate FAK [91]. Activation of PI3-kinase/Akt pathway is another signaling event initiated by integrins. Activation of FAK may act upstream in the activation of PI3-kinase/Akt pathway [92, 93] and inactivation of GSK-3*β* [94]. In ARVM, adenoviral-mediated expression of *β*1A integrin increased FAK phosphorylation at both Tyr397 and Tyr576 residues without affecting expression of FAK. Inhibition of MMP-2 using SB3CT or TIMP-2 significantly increased FAK phosphorylation (Tyr-397 and Tyr-576). On the other hand, active MMP-2 significantly inhibited FAK phosphorylation [62]. *In vivo*, chronic stimulation of *β*_1_-AR impaired FAK signaling during early compensated mitral regurgitation in dogs [95]. In cat atrial myocytes, binding of laminin to *β*1 integrins inhibited adenylyl cyclase activity via the involvement of FAK/PI3-kinase/Akt pathway [96]. Our laboratory has provided evidence that expression of *β*1A integrin or inhibition of MMP-2 inhibits *β*-AR-stimulated activation of GSK-3*β*. On the other hand, active MMP-2 protein increased GSK-3*β* activity. Inhibition of PI3-kinase using wortmannin reversed the effects of *β*1 integrins on GSK-3*β* activity and inhibited the protective effect of *β*1 integrins [51]. Activation of JNKs and expression of MMP-2 were significantly greater in the myocardium of *β*1-integrin-deficient mice when compared to wild type following chronic *β*-AR stimulation [9]. It is likely that MMP-2 disrupts the anti-apoptotic signals initiated by *β*_1_ integrin engagement, resulting in the activation of a JNK-dependent mitochondrial death pathway. Of note, extracellular ubiquitin also inhibited *β*-AR-stimulated activation of GSK-3*β*, and inhibition of PI3-kinase using wortmannin reversed the protective effects of extracellular ubiquitin [52], suggesting the possibility that extracellular ubiquitin may signal via *β*1 integrins. Co-immunoprecipitation studies demonstrated physical association of MMP-2 with *β*1 integrins in ARVM. *β*-AR stimulation increased the level of interaction between these two proteins, while inhibition of MMP-2 using SB3CT or stimulation of *β*1 integrin signaling using laminin inhibited *β*-AR-stimulated interaction of MMP-2 with *β*1 integrins [61]. It is likely that physical interaction of MMP-2 with *β*1 integrins may interfere with the survival signals induced by *β*1 integrins, leading to apoptosis.

## 5. Conclusion

**Figure 1:**
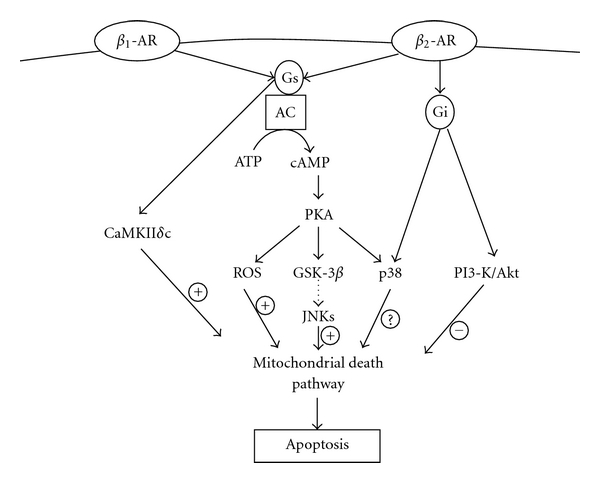
Summary diagram illustrating signaling pathways involved in *β*-AR-stimulated cardiac myocyte apoptosis. AR: adrenergic receptors; JNKs: c-Jun-N-terminal kinase; GSK-3*β*: glycogen synthase kinase-3*β*; ROS: reactive oxygen species; PKA: protein kinase A; AC: adenylyl cyclase; Gs: stimulatory G protein; Gi: inhibitory G protein; ATP: adenosine triphosphate; cAMP: cyclic adenosine monophosphate; CaMKII*δ*: calcium calmodulin kinase II*δ;* PI-3K: phosphatidylinositol 3-kinase.

**Figure 2:**
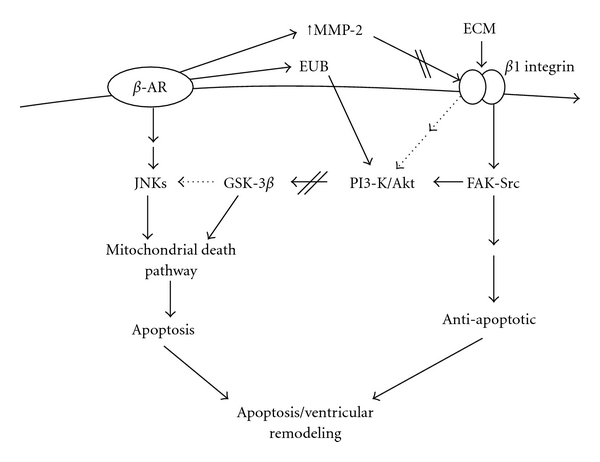
Summary diagram illustrating *β*-AR signaling and the role of *β*1 integrins in *β*-AR-stimulated cardiac myocyte apoptosis and myocardial remodeling. ECM: extracellular matrix proteins; EUB: extracellular ubiquitin; FAK: focal adhesion kinase.

Catecholamines play an important role in remodeling of the heart, when the heart is subjected to pathophysiological stressors. *β*_1_-AR and *β*_2_-AR coupled to G*α*s exert a pro-apoptotic action via a cAMP-dependent mechanism which appears to involve mitochondria and ROS and is associated with the activation of JNK and GSK-3*β*. Pro-apoptotic action of *β*_1_-AR may involve activation of CaMKII. Conversely, *β*_2_-AR coupled to Gi exerts an anti-apoptotic action which is mediated by PI3-kinase/Akt (Figure 1). Elucidation of processes that can shift the balance from apoptosis to cell survival during chronic *β*-adrenergic stimulation may have important clinical implications. Identification of molecular targets involved in the activation of JNKs and GSK-3*β* following *β*-AR stimulation and understanding how these kinases activate mitochondrial death pathway may provide new targets for prevention of heart failure. *β*1 integrins play an important role in chronic *β*-AR-stimulated cardiac myocyte apoptosis and myocardial remodeling via the involvement of FAK and PI3-kinase/Akt pathways (Figure 2). The structural changes in myocardial ECM are considered to play an important role in the modulation of myocardial function and in the progression to heart failure. Analysis of components of ECM, including laminin, collagen type I and IV, and fibronectin, may provide insights into the regulation of heart function by *β*1 integrins. Further studies aimed at determining the molecular mechanism by which interaction of MMP-2 with *β*_1_-integrins affects *β*-AR-stimulated apoptosis in cardiac myocytes may have important implications for the regulation of myocyte survival.
